# Competitiveness and the Process of Co-adaptation in Team Sport Performance

**DOI:** 10.3389/fpsyg.2016.01562

**Published:** 2016-10-10

**Authors:** Pedro Passos, Duarte Araújo, Keith Davids

**Affiliations:** ^1^CIPER, Faculdade de Motricidade Humana, Universidade de LisboaLisboa, Portugal; ^2^Centre for Sports Engineering Research, Sheffield Hallam UniversitySheffield, UK

**Keywords:** competitive behavior, team sports, interpersonal coordination, affordances, constraints

## Abstract

An evolutionary psycho-biological perspective on competitiveness dynamics is presented, focusing on continuous behavioral co-adaptations to constraints that arise in performance environments. We suggest that an athlete’s behavioral dynamics are constrained by circumstances of competing for the availability of resources, which once obtained offer possibilities for performance success. This defines the influence of the athlete-environment relationship on competitiveness. Constraining factors in performance include proximity to target areas in team sports and the number of other competitors in a location. By pushing the athlete beyond existing limits, competitiveness enhances opportunities for co-adaptation, innovation and creativity, which can lead individuals toward different performance solutions to achieve the same performance goal. Underpinned by an ecological dynamics framework we examine whether competitiveness is a crucial feature to succeed in team sports. Our focus is on intra-team competitiveness, concerning the capacity of individuals within a team to become perceptually attuned to affordances in a given performance context which can increase their likelihood of success. This conceptualization implies a re-consideration of the concept of competitiveness, not as an inherited trait or entity to be acquired, but rather theorizing it as a functional performer-environment relationship that needs to be explored, developed, enhanced and maintained in team games training programs.

## Introduction

In the current research literature there are three different approaches to understanding competitiveness: (i) a psychological perspective where competitiveness is conceptualized as an innate drive and viewed as a personality trait (Kayhan, 2003); (ii) another psychological view where competiveness is understood as a dynamical mental state which drives a performer toward excellence sustained by social comparisons to be better than others (Jones, 2015); and (iii) an evolutionary biological perspective where competitiveness is seen at the behavioral level as the ability to use resources in competition with others (Baldauf et al., 2014).

From the theoretical perspective of ecological dynamics, competitiveness can be conceptualized as a constraint on sports performance which influences emergence of a performer’s competitive behaviors. At an ecological level competitiveness is a constraint, resulting from the confluence of environment, task and personal constraints, which can be managed during training, for instance, with added rules (e.g., receive the ball while running), spatial-temporal constraints (e.g., short interpersonal distances), or manipulated pressure (e.g., technical and tactical similarity among opponents). But a key issue to enhance competitiveness is that these tasks constraints need to be manipulated to ‘push’ players beyond current performance levels, otherwise increasing competitiveness has little functionality in the representative practice contexts.

Competitiveness in a performance context is a constraint that creates affordances [i.e., possibilities for action, (Gibson, 1979)]. Consequently, sport practice programs provide an opportunity to simulate important performance sub-phases where such affordances can be perceived. Here we propose an interaction between the psychological and biological perspectives, in the form of an evolutionary psycho-biological framework to explore the idea that competitiveness can be characterized at the individual-environment level in behavioral dynamics. Continuous co-adaptations of individuals to constraints arise from situational factors which bound each individual’s competitive behaviors.

This theoretical rationale sharply contrasts with considering competitiveness as a psychological entity to be gained or as an inherited trait. Rather it can be viewed at the level of the integrated performer-environment system, as a functional relationship that needs to be explored, enhanced and maintained in sport practice programs.

### How Intrateam Competition Enhances ‘Fitness’ for a Performance Environment

The relevance of situational factors, such as performance standards or the number of competitors involved in a collective system, can influence an athlete’s competitive behaviors in sport (Garcia et al., 2013).

In discussing competitiveness there is a need to focus on the interaction between players in the same group, competing for selection by a coach, for example. As noted earlier, competitiveness (from a biological perspective) can be defined as the ability to use resources in competition with others (Baldauf et al., 2014). This definition supports the need to create, within the same team, an ‘interteam’ environment (e.g., small-sided and conditioned games, designing task constraints representative of specific sub-phases of competitive performance environments, e.g., 2v1; 3v2) which can increase intrateam competitiveness. By creating these competitive environments within squads of athletes, two categories of resources are uncovered, for which individuals have to compete: (i) *intrateam resources* which lead to competition between teammates (e.g., the development of technical and tactical skills to struggle for selection at a development academy or for a position in the senior squad); and (ii), *interteam resources* which lead to enhanced competitive behaviors against opponents.

Thus, each athlete’s abilities to seek resources to function competitively will lead to the acquisition of psycho-physical, social and emotional resources over a long time scale (e.g., enabling athletes to become more functional in performance), enhancing their capacity to compete and gain selection, key roles, and status within a squad. An intrateam focus on competitiveness is needed in coaching, not driven by external comparisons for their own sake but to understand and re-define an individual’s ‘fitness’ to compete in team sports. The term ‘fitness’ is not used as in the conventional way in sports training to signify a level of physical conditioning. Rather in this paper it has a connotation from the evolutionary sciences which examines the *functionality* of a relationship between an organism and its environment (Kauffman, 1995). A fitness landscape captures a range of behaviors that define how an organism can utilize affordances to enhance its functionality (e.g., successfully achieving goals and subgoals) in competing with other members of its species (intra-species competitiveness) and with other species (inter-species competitiveness). Enhancing the ‘fitness’ of athletes to achieve resources and performance goals, enables them to compete for resources that allow them to perform more successfully (i.e., overcome opponents, support teammates, win in competition, earn sponsorships, achieve better professional contracts) through exploiting similar processes of co-adaptation (Davids et al., 2008).

### The Process of Co-adaptation

In nature, different biological systems have developed tools which enhance their competitiveness within their own species through the process of continuous co-adaptation to arising constraints. This concept is also influential in understanding how the process of competitiveness between and within athletes in sport can be functional for enhancing development, learning, and performance. Although evolution, learning, development, and performance have different timescales, their dynamical processes are predicated on the same principles. The key point in ecological dynamics is that the same principles underlie system dynamics, regardless of timescales of emergence (Newell et al., 2001).

In ecological dynamics, the term ‘fitness’ at an evolutionary scale of analysis can be helpful in describing how functionally adapted an individual member of a species is to the affordances in an econiche. Species change due to continuous interactions with other species and with their environment, and the dynamical process of continuous co-adaptation drives the co-evolution of functional behaviors (Kauffman, 1995). At the heart of these continuous interactions between species and environmental constraints, is a competition between biological organisms for resources noted earlier. In this way co-adaptation is the engine of evolutionary change. However, it is possible to characterize the term ‘interaction’ in two ways: (i) if there is no incentive to change, two competing species might keep their distance from each other and each population would evolve toward a steady state (no competitiveness); or (ii), in contrast, affordances provide opportunities for specific behaviors to emerge, for instance to compete for resources which enhance functionality (competitiveness). Competing for resources in one population might open the possibility for new affordances, due to the emergence of new skills leading to adaptive behaviors (Kauffman, 1995). These enhanced capacities within individual members of a species provide an ‘optimal grip’ on the specific ‘form of life’ that surrounds an individual athlete in sport, including the social and cultural ‘climate’ during practice and training (Rietveld and Kiverstein, 2014; Davids et al., 2016).

The process of co-adaptation drives an organism’s relations with its environment in different directions, some of which may enhance its fitness in a performance environment, whereas others may lead to performance decrements and ‘extinction’ in the form of lack of competitiveness.

The utilization of affordances is a major feature of each individual’s capacity to co-adapt to task and environmental constraints through competition which coaches can facilitate.

As mentioned earlier, the term affordance refers to action possibilities, and to perceive an affordance is to perceive how one could act with respect to a performance environment in sport. However, affordances are neither external properties of an environment, nor are they mentalistic properties of the mind. Rather, affordances are relational properties of an individual-environment system and capture the action specific relations that exist between the action capabilities of an individual performer and the action relevant properties of the substances, surfaces, objects, others and events of a performance environment. In other words, affordances capture the “fit” between an individual and environment (Gibson, 1979).

In order to utilize affordances, individuals allocate different resources to enhance their competitive capacity: some may invest in physical resources (e.g., velocity, strength, flexibility), others may invest in perceptual abilities [e.g., increase the speed of gaze (scanning) patterns to anticipate threats from opponents]. Some individual organisms adopt risky behaviors (e.g., being more creative and playing with flair) than others, who prefer to perform conservatively, avoiding risky decisions. These different behaviors will shape the overall competitiveness of a group. Thus, competitiveness enhances innovation and creativity which provides individuals with different performance solutions for achieving the same goal (Kuperberg, 2003).

### Co-adaptation and Ecological Dynamics in Sport

Previous research has suggested that continuous attacker-defender interpersonal interactions in team sports, can be considered as emerging from a dyadic (1 vs. 1) sub-system, evolving by alternating between periods of stability and variability (Passos et al., 2009, 2013). In these team game dyadic systems, defenders compete with attackers to maintain system stability (remaining between the attacker and the goal/try line/basket), as attackers seek to de-stabilize it (Passos and Davids, 2015; Shafizadeh et al., 2016). As a consequence, the ‘fitness’ of performers in adapting to the changing competitive system can become more demanding. There is a tightening of space-time constraints which shorten the time for actions (Araújo et al., 2013) due, for instance, to a decrease in values of interpersonal distance between players. As the competitive sport system evolves there is a concomitant need for athletes to engage in exploratory behaviors to seek and establish functional movement solutions to satisfy the changing constraints of competitive performance (Davids et al., 2012).

In team sports the capacity to co-adapt behaviors in seeking affordances to utilize during competitive performance are predicated on two sorts of interpersonal coordination processes: intrateam coordination and interteam coordination. Intrateam coordination is supported by cooperation among players of the same team, where the patterns formed (e.g., geometric shapes formed by players’ relative position) are characterized as preferred system states (Warren, 2006), offering specific affordances for those involved. During competitive performance in team games, the decreasing of interpersonal distance between competing players can disturb intrateam coordination patterns, continually demanding co-adaptive behaviors between performers to support different behavioral solutions to overcome opposition strategies. This aspect of co-adaptation between performers emphasizes the need for cooperation within a collective system in order to remain competitive. The emergence of different behavioral solutions can signify that previous preferred system states may no longer have been functional. That is, affordances available for utilizing an intrateam pattern of coordination may no longer have been available. As a consequence, the players need to reorganize into functional system states, as other affordances become available. In co-adapting to opponents, performers may need to transit from one intrateam coordination pattern to another, since ‘new’ patterns of co-adaptive cooperation open for ‘new’ affordances, from a landscape of affordances (Rietveld and Kiverstein, 2014) which continuously evolve according to competitive dynamics.

Additionally, performers need to adapt to competitive constraints by exploiting interteam coordination processes, i.e., attacker-defender interpersonal coordination tendencies. Theoretically, interteam coordination tendencies remain relatively stable when both sides play within the rules and the ‘spirit’ of the game. Further, there are some rare instances when teams are happy to share a tied game and do not need to compete as they would normally for the same resources, for instance, to penetrate defensive space on field as they would normally, or to fight for ball possession. Therefore, competing sport teams can be conceptualized as components of a dynamical system which can display competing and cooperative tendencies (Davids et al., 1994; McGarry et al., 2002).

However, from the range of component variables that might characterize a dynamical system there is a subset of variables known as ‘essential variables’^1^ (Ashby, 1960; Kauffman, 1993). For instance, in sport such systems may involve two competing players and the variables might include physiological states, emotional states, but also technical and tactical skills. In an attacker-defender system which remains in a steady state, the values of these ‘essential variables’ must be kept within specific bounded ranges. When for some reason system constraints lead to a change in the values of one or more essential variables pushing them beyond the boundaries, system stability might be disturbed. Then the system might be poised to ‘jump’ to another preferred state, where the essential variables are maintained within other boundaries (or not). We argue that these ‘jumps’ are changes in the performer-environment system that occur after perceiving and realizing a new affordance, here conceived as an attractor. Ashby (1960) suggested that the ’fittest’ (most functional) attractors in the landscape of affordances were preferred system states (Ashby, 1960; Kauffman, 1993). Relating this idea from theoretical biology back to the example of attacker-defender dyads in rugby union, if the values of system essential variables (e.g., each player’s running line velocity) remain within specific boundary limits (i.e., both contributing to a stabilization in the difference in running line velocity values) the system will remain in a current state of stability, which obviously favors the defender. However, an increase in value of the attacker’s velocity, and a stabilization or decrease in the value of a defender’s running line velocity, will drive an attacker-defender system to transit to another preferred system state (another attractor in the landscape), providing an advantage for the attacker (Passos et al., 2008). This is a core idea in the paper: Changes in system essential variables (due to a dynamical constraints of a competitive performance environment) will ‘push’ the entire system to another preferred state that exists in the competitive performance landscape. Jumps/transitions between preferred system states *only* occur due to changes in the values of system essential variables, which in turn are influenced by key constraints of a competitive performance environment. It is important to note that changes in values of essential variables can be due to the use, when competing, of ‘new’ individual resources (e.g., an increase in the acceleration profile or strength gains or adoption of an innovative ‘new’ dribbling technique in team sports), which may only emerge as a consequence of the co-adaptations to task and environmental constraints. This is how pedagogical practice and sport science support can greatly enhance the competitive behavior of individual athletes, by designing affordance landscapes in training enhancing competitiveness to ensure that performers can seek and exploit resources beyond individual limits.

### Competitiveness and the Implications for Skill Acquisition

Continuous co-adaptations, from developmental athlete to expert performer status, continually emphasize the need for individuals to train to adapt to the dynamic constraints of a competitive performance environment. Co-adaptations demanded by teammates and by coaching and sport science staff provide a platform of competitiveness for harnessing the competitive behavior of an individual to improve his/her own performance standards and enhance their competitive ’fitness’ in the performance environment.

Such a conceptualization suggests that *skill acquisition* needs to be considered as *skill adaptation*, continuously constrained by key features of a performer-environment system (e.g., opponent skill levels; player perceptual systems; player technical skills; tactical performance behaviors; Araujo and Davids, 2011). An implication of harnessing competitiveness in practice is that the mutual and reciprocal interaction of the player-environment system enhances the attunement of performers to available information which can be used to functionally regulate their actions, during skill acquisition (Davids et al., 2012). During interactions with surrounding performers each individual learns to perceive new affordances in a competitive environment according to their evolving skill. In other words, skill acquisition leads to changes in properties of a specific competitive environment to which each individual’s perceptual systems become attuned (Araújo et al., 2013; Passos and Davids, 2015).

During the course of action ongoing perceptual regulation sustains an individual performer’s adaptive behaviors to satisfy specific task constraints, for instance the time needed to reduce the distance to an opponent (Davids et al., 2012). It needs to be noted that a performer’s adaptive behaviors tend to create fluctuations in interpersonal coordination tendencies. Such fluctuations do not exist *a priori*, since they emerge molded by specific task constraints (Davids et al., 2012), such as the values of interpersonal distances to an opponent (Passos et al., 2008; Shafizadeh et al., 2016); or the interpersonal angle between ball carrier, the location of the goal and the closest defender (Vilar et al., 2013, 2014). Fluctuations provide information for affordances to which performers need to become attuned during practice and performance. These fluctuations only occur within critical regions where performance behaviors are no longer independent from other adjacent individuals (i.e., teammates; opponents), and each individual has to compete for available resources in order to succeed.

The level of competitive behavior varies considerably across individuals in space and time (Baldauf et al., 2014). Some players display more competitive behaviors in key performance areas, for example closer to their own goal area, whereas other individuals become more competitive closer to the opposition’s goal area. Some players become highly competitive at selected time points, for example in different periods of a match, whereas others are highly competitive as soon as a match begins. In training these individual differences need to be explored and enhanced through designing an affordance landscape for individuals at different expertise levels. In competitive environments performers need to be attuned to affordances that support preferred behavioral states which satisfy constraints in dynamic contexts where unpredictability is ubiquitous.

## Conclusion

The acquisition of new skills requires exploratory behaviors on the part of each athlete who has to assemble unique functional movement solutions to satisfy particular task constraints. The perceptual-motor landscape of each individual changes as a consequence of new experiences and the acquisition of new skills. This aspect of skill acquisition means that players develop skills to enable them to compete for available resources (e.g., space-time gaps to perform key actions successfully; preventing opponents from dictating play or to de-stabilize a dyadic system formed with an adjacent opponent). Understanding practice designs for exploiting co-adaptive moves will help athletes and sports teams, as complex adaptive systems, to harness competitiveness in an intrinsic way so that each player drives the adaptations needed to continually re-define their ‘fitness’ for an ‘optimal grip’ on a form of life in sport performance (Davids et al., 2016).

## Author Contributions

PP contributes with the skeleton and first draft of the chapter, more especifically with the issues related with co-adaptation in collective behaviors as team sports. DA contributes to the theoretical issues regarding the ecological dynamics approach as a framework to competitive practice designs. KD contributes for the theoretical issues related with the constraints led approach.

## Conflict of Interest Statement

The authors declare that the research was conducted in the absence of any commercial or financial relationships that could be construed as a potential conflict of interest.

## Footnotes

1 The term ‘essential variables’ can be equated to ‘control parameter,’ previously used in the literature (see Passos et al., 2008 as an example). The terms can relate to each other due to the fact that when achieved a critical ‘value’ the system jump to a new performance state.
